# The Impact of Adding a Physician Assistant to a Critical Care Outreach Team

**DOI:** 10.1371/journal.pone.0167959

**Published:** 2016-12-12

**Authors:** Hayley B. Gershengorn, Yunchao Xu, Carri W. Chan, Mor Armony, Michelle N. Gong

**Affiliations:** 1 Division of Critical Care Medicine and Department of Neurology; Albert Einstein College of Medicine, Montefiore Medical Center; Bronx, New York, United States of America; 2 Department of Information, Operations, and Management Sciences; New York University Stern School of Business; New York, New York, United States of America; 3 Division of Decision, Risk, and Operations; Columbia Business School; New York, New York, United States of America; 4 Division of Critical Care Medicine and Department of Epidemiology and Population Health; Albert Einstein College of Medicine, Montefiore Medical Center; Bronx, New York, United States of America; Azienda Ospedaliero Universitaria Careggi, ITALY

## Abstract

**Rationale:**

Hospitals are increasingly using critical care outreach teams (CCOTs) to respond to patients deteriorating outside intensive care units (ICUs). CCOT staffing is variable across hospitals and optimal team composition is unknown.

**Objectives:**

To assess whether adding a critical care medicine trained physician assistant (CCM-PA) to a critical care outreach team (CCOT) impacts clinical and process outcomes.

**Methods:**

We performed a retrospective study of two cohorts—one with a CCM-PA added to the CCOT (intervention hospital) and one with no staffing change (control hospital)—at two facilities in the same system. All adults in the emergency department and hospital for whom CCOT consultation was requested from October 1, 2012-March 16, 2013 (pre-intervention) and January 5-March 31, 2014 (post-intervention) were included. We performed difference-in-differences analyses comparing pre- to post-intervention periods in the intervention versus control hospitals to assess the impact of adding the CCM-PA to the CCOT.

**Measurements and Main Results:**

Our cohort consisted of 3,099 patients (control hospital: 792 pre- and 595 post-intervention; intervention hospital: 1114 pre- and 839 post-intervention). Intervention hospital patients tended to be younger, with fewer comorbidities, but with similar severity of acute illness. Across both periods, hospital mortality (p = 0.26) and hospital length of stay (p = 0.64) for the intervention vs control hospitals were similar, but time-to-transfer to the ICU was longer for the intervention hospital (13.3–17.0 vs 11.5–11.6 hours, p = 0.006). Using the difference-in-differences approach, we found a 19.2% reduction (95 confidence interval: 6.7%-31.6%, p = 0.002) in the time-to-transfer to the ICU associated with adding the CCM-PA to the CCOT; we found no difference in hospital mortality (p = 0.20) or length of stay (p = 0.52).

**Conclusions:**

Adding a CCM-PA to the CCOT was associated with a notable reduction in time-to-transfer to the ICU; hospital mortality and length of stay were not impacted.

## Introduction

Critical illness can arise anywhere in the hospital, emergency department, or even prior to hospital arrival. Moreover, critical illness is often optimally managed when treatment is initiated early.[1, 2] For these reasons, numerous quality groups advocate for rapid response/medical emergency teams (RR/METs) to attend quickly to patients experiencing clinical deterioration outside the intensive care unit (ICU).[3–5] While data on their impact is mixed,[6–10] these teams are increasingly common across U.S. hospitals and internationally.[11]

Who and how many clinicians should staff these RR/METs to make them most clinically- and cost-effective is not known. Recommendations are that team composition be determined by each “institution’s resources and needs.”[5] Published literature suggests many models—including intensivists, hospitalists, housestaff, non-physician-providers and critical care nurses—are used.[12–16] How one team structure compares to another and the impact of including clinicians with different backgrounds is not well studied. In a single center study of RR/METs led by intensivists versus resident physicians, there was no difference in patients’ progression to cardiac arrest, need for ICU admission, or hospital mortality;[13] yet, still more work needs to be done to understand whether this is the “right” RR/MET structure or if other configurations may be more effective and/or just as effective, but with lower costs.

The role of physician assistants with training/experience in critical care medicine (CCM-PAs) has received some attention in the literature.[17–19] This work has focused primarily on in-ICU staffing[20–22] and has found that employing CCM-PAs as replacements for physicians-in-training can be a safe and effective way to expand the workforce in critical care. Whether replacing an existing team member or adding a CCM-PA to an RR/MET could provide similar benefits or, conversely, might be detrimental is unknown. Specifically, having the CCM-PA take on some of the RR/MET work (e.g. performing procedures, evaluating and managing deteriorating patients awaiting ICU admission, etc.) might reduce delays in ICU admission and, possibly, improve patient outcomes; however, adding a CCM-PA could negatively impact care by delaying assessment by a different provider.

The two tertiary, academic hospitals at Montefiore Medical Center have had a critical care outreach team (CCOT)[11, 23, 24] composed of an intensivist and critical care fellow since 1995. In 2013, a CCM-PA was added to the CCOT at one of the hospitals. Staffing changes are common in healthcare systems, but the impact of such interventions is rarely rigorously evaluated. In this study, we sought to test the hypothesis that adding the CCM-PA would result in better clinical and process (e.g., delays in ICU transfer) outcomes for patients evaluated by the CCOT.

## Methods

We conducted a retrospective cohort study of all patients seen by the adult CCOT at one of the two academic tertiary care facilities (one with 620 total and 46 critical care beds, the other, 396 total and 33 critical care beds) at Montefiore Medical Center in the Bronx, New York. The larger hospital had 24,878 hospital admissions with an overall hospital mortality of 2.2% in 2014; the smaller, 22,471 admissions and a 1.7% mortality rate. The role of the CCOT in these institutions is to respond to medical emergencies and cardiac arrests outside of the emergency room and ICUs, to evaluate new critical care consultations in the emergency department, on the wards, and in the post-anesthesia care unit, and to provide procedural assistance (e.g., vascular access) throughout the hospital. All patients with acute deterioration or unplanned ICU transfer require CCOT evaluation prior to ICU admission; most surgical patients enter the ICU in the immediate post-operative period without CCOT evaluation. CCOTs began in both institutions in 1995 and were staffed since inception by a board certified intensivist and critical care fellow 24 hours/day, 7 days/week. Starting on March 17, 2013, a CCM-PA was gradually added (first during the daytime on weekdays, then weekends, and then overnight) to the CCOT at the larger hospital (intervention hospital) with complete PA coverage 24 hours/day, 7 days/week by December 16, 2013; to allow for a run-in, we used January 5, 2014 as the initiation date for the post-period analyses. During this time, Montefiore Medical Center employed 37 CCM-PAs; a different CCM-PA per 12.5 hour shift staffed the CCOT. The PA’s role included assisting in new patient evaluations, stabilizing/monitoring deteriorating patients not yet in the ICU, and performing procedures (e.g., central venous catheterizations). Prior to the addition of the CCM-PA, all of these tasks were performed by the board certified intensivist and critical care fellow who comprised the CCOT. During the same time, there was no addition of a PA to the control hospital because of limited resources. No other changes were made to the staffing structures or roles/responsibilities of these CCOTs during this time.

### Cohort

**Fig 1:**
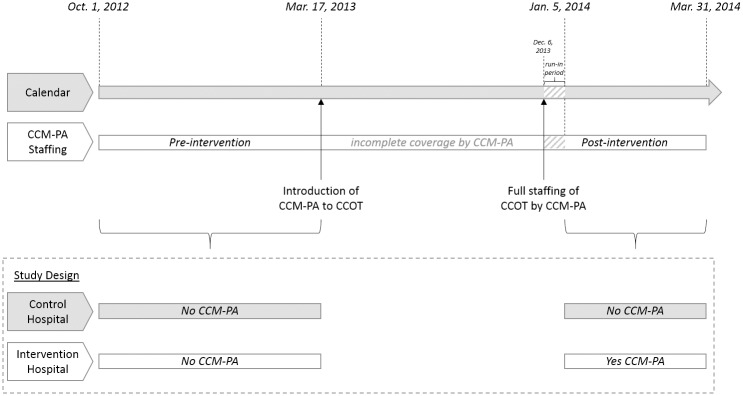
Timeline of study. CCM-PA: Critical Care Medicine Physician Assistant; CCOT: Critical Care Outreach Team.

Our cohort consisted of all patients for whom CCOT consultation was requested in each of the two hospitals from October 1, 2012 –March 16, 2013 (pre-intervention) or January 5 –March 31, 2014 (post-intervention); patients consulted upon from March 17, 2013 –January 4, 2014 were excluded as there was incomplete CCM-PA staffing during this period (Fig 1). Patients were excluded if consultations were clearly requested solely for procedural assistance. Montefiore Medical Center has a separate pediatric CCOT system, thus, the vast majority of CCOT consultations were on adults (≥21 years-old); however, patients were not specifically excluded based on age.

Clinical data were available from the electronic medical record and were accessed using Clinical Looking Glass.[25] Patient-level characteristics included demographics (age, race/ethnicity, gender, insurance provider), chronic illness burden (determined by Elixhauser comorbidities[26]), primary admission diagnosis category (determined by International Classification of Diseases, 9^th^ Edition coding on hospital discharge), severity of illness (determined by Laboratory-based Acute Physiology Score, LAPS,[27] and Sequential Organ Failure Assessment score, SOFA[28]) at the time of CCOT evaluation, need for ICU admission, goals of care (full care including resuscitation/intubation if needed versus orders not to resuscitate and/or intubate) at the time of CCOT evaluation, timing of hospital admission (weekend versus weekday), and timing of CCOT evaluation (night—7pm-6:59am versus day—7am-6:59pm, day of week, month). Outcomes were time-to-transfer to the ICU (time from the initial request for CCOT evaluation to when the first set of vital signs was recorded in the ICU, TtT_ICU_) for patients admitted to the ICU, hospital mortality, and hospital length of stay (LOS).

### Statistical Analysis

Standard summary statistics were used to compare baseline characteristics and unadjusted outcomes between patients admitted to the intervention and control hospitals (both pre- and post-intervention). A difference-in-differences approach was used to evaluate the impact of the intervention (introducing the CCM-PA to the CCOT) after adjustment for potential confounders.[29–32] This methodology uses a multivariable regression modelling technique with an interaction term for “time period” (pre- versus post-intervention) and “hospital” (intervention versus control) to allow assessment of the independent association of the intervention with an outcome. In contrast to more standard before-after studies, the use of the control hospital enables control for unobservable temporal trends in patient severity that might impact outcomes.

**Fig 2:**
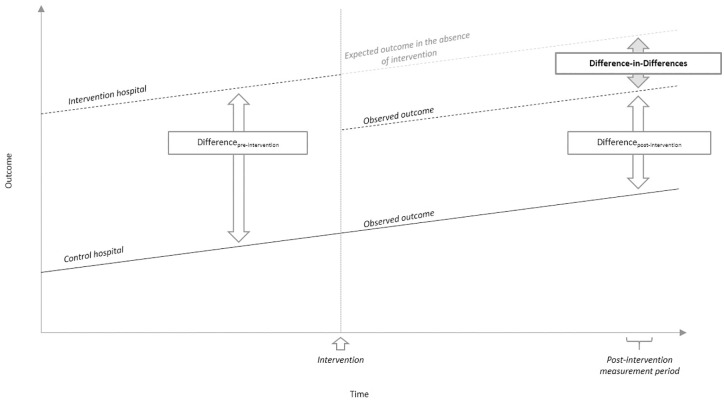
Pictorial representation of difference-in-differences methodology. Assumptions of the methodology include: (1) single intervention as depicted by the ⇧ and (2) same baseline trends in outcome as demonstrated by parallel outcomes prior to the intervention. The difference-in-differences is equal to the difference between the intervention and control hospitals pre-intervention (difference_pre-intervention_) minus the difference between the intervention and control hospitals post-intervention (difference_post-intervention_).

Two underlying assumptions are central to the difference-in-difference technique: that (1) no interventions other than that being studied occurred during the study period to one hospital differently than the other and (2) trends in outcomes for the control and intervention hospitals were similar pre-intervention (Fig 2). As aforementioned, no other changes were made to the CCOT at either hospital during this period. To test the second assumption, we used multivariable regression modeling for only patients admitted during the pre-intervention period; the model included the date of CCOT evaluation, the hospital (control versus intervention), and all other patient-level covariates. An insignificant association of hospital (control versus intervention) and outcome would indicate similar baseline trends for that outcome.

Separate multivariable difference-in-difference regression models were constructed to evaluate the association of the CCM-PA CCOT intervention and TtT_ICU_, hospital mortality, and hospital LOS; TtT_ICU_ and hospital LOS were logarithmically transformed. In addition to the inclusion of hospital (intervention versus control), time period (pre- versus post-intervention), and their interaction term, all measured patient-level characteristics were included in each model as covariates. Finally, four additional models were constructed for TtT_ICU_ using three-way interactions (hospital*time period*“stratified groups” with “stratified groups” defined separately by age </≥ 65, Elixhauser comorbidities </≥ 2, diagnosis of infectious disease vs not, and diagnosis of respiratory illness vs not) to assess whether adding the CCM-PA to the CCOT affected subgroups differentially.

Statistical analyses were performed using R 3.2.3 (The R Foundation for Statistical Computing, Vienna, Austria) and Microsoft Excel (Microsoft, Redmond, WA). Patient information was de-identified prior to analysis. Institutional Review Board approval was obtained from Albert Einstein College of Medicine (#2014–3190).

## Results

Our cohort consisted of 3,099 patients (control hospital: 792 pre- and 482 post-intervention; intervention hospital: 1114 pre- and 711 post-intervention, Table 1). Patients evaluated by the CCOT at the intervention hospital tended to be younger (62.7 years-old pre- and 61.3 post-intervention vs 67.4 and 66.3 in the control hospital, p<0.01), more commonly African-American (38.8% pre- and 38.0% post-intervention vs 30.2% and 30.8%, p<0.01), and more likely to have Medicare/Medicaid insurance (64.5% pre- and 65.8% post-intervention vs 59.0% and 60.5%, p<0.01). They tended to have fewer comorbidities (Elixhauser comorbidities: 4.3 pre- and 4.3 post- intervention vs 4.5 pre- and 4.6 post-, p<0.01) and lower LAPS (59.2 pre- and 55.4 post-intervention vs 60.1 pre- and 59.9 post-, p = 0.037), but higher SOFA (3.9 pre- and 3.7 post-intervention vs 3.7 pre- and 3.6 post-, p<0.01) at the time of CCOT consultation. While LAPS and SOFA scores both measure acuity, their use of vital signs and laboratory data differ.

**Table 1:** Baseline characteristics of cohort stratified by hospital and time period.

	Control Hospital			Intervention Hospital			Control vs. Intervention
	Pre-PA on CCOT	Post-PA on CCOT	p-value*	Pre-PA on CCOT	Post-PA on CCOT	p-value*	p-value^†^
Number of Patients	792	482		1114	711		
Age (years)	67.4	66.3	0.28	62.7	61.3	0.098	<0.01
Male gender, (%)	44.2%	43.2%	0.76	49.0%	47.1%	0.43	<0.01
Race, (%)			<0.01			<0.01	<0.01
White	33.2%	32.1%		19.2%	16.3%		
Black/African American	30.2%	30.8%		38.8%	38.0%		
Asian	1.3%	2.0%		2.2%	1.9%		
Asian/Black-African Amer.	0.3%	0.2%		0.3%	0.4%		
Asian/White	0.4%	0.0%		0.1%	0.0%		
Black-African Amer./White	0.1%	0.2%		0.4%	0.4%		
Hawaiian/Pacific Islander	0.0%	0.0%		0.2%	0.5%		
Multiracial, Other	28.3%	29.4%		30.1%	33.0%		
Declined to answer	6.1%	5.2%		7.7%	8.7%		
Unavailable	0.1%	0.2%		0.9%	0.7%		
Ethnicity, (%)			0.56			0.035	<0.01
Hispanic or Latino	33.7%	30.6%		35.7%	40.0%		
Not Hispanic or Latino	58.5%	62.2%		61.0%	55.3%		
Declined to answer	7.7%	7.1%		2.3%	3.8%		
Unavailable	0.1%	0.2%		1.0%	0.9%		
Insurance, (%)			0.84			0.41	<0.01
Medicare/Medicaid	59.0%	60.5%		64.5%	65.8%		
Private Pay	40.3%	38.8%		34.8%	34.0%		
Self Pay	0.8%	0.7%		0.6%	0.2%		
Elixhauser Comorbidity Index	4.5	4.6	0.35	4.3	4.3	0.96	<0.01
Severity of Illness Scores							
SOFA, time of consult	3.7	3.6	0.51	3.9	3.7	0.053	<0.01
LAPS, hospital admission	50.8	51.5	0.67	46.8	46.3	0.75	<0.01
LAPS, time of consult	60.1	59.9	0.94	59.2	55.4	0.020	0.037
Admitting Diagnosis, (%)			<0.01			0.004	<0.01
Cancer/tumor	5.2%	5.0%		7.9%	13.0%		
Cardiovascular	12.6%	9.6%		14.3%	10.3%		
Endocrine/Metabolic/Renal	9.1%	9.4%		9.3%	7.7%		
Gastrointestinal	10.1%	10.4%		8.6%	9.1%		
Gynecologic	2.1%	1.3%		0.3%	0.2%		
Hematologic	2.4%	1.8%		2.1%	3.7%		
Infectious Disease	33.7%	42.5%		33.8%	33.6%		
Neurologic	10.1%	8.1%		11.9%	11.4%		
Respiratory	11.9%	9.7%		9.2%	8.1%		
Other	2.8%	1.8%		2.4%	2.6%		
DNR, time of consult, (%)	0.9%	1.1%	0.96	0.7%	1.3%	0.26	0.55
Hospital Admission Data							
Weekend Admission, (%)	25.0%	27.7%	0.25	24.6%	25.6%	0.60	0.46
Admitted from the ER, (%)	86.1%	89.4%	0.076	79.5%	82.0%	0.19	<0.01
Consult Information							
Admitted to the ICU following consultation, (%)	0.6%	0.2%	0.16	0.5%	0.5%	0.85	0.74
Timing							
Overnight^‡^, (%)	41.5%	40.8%	0.79	44.3%	40.9%	0.13	0.35
Day of the Week, (%)			<0.01			0.58	0.92
Monday	11.6%	17.1%		14.8%	14.1%		
Tuesday	17.2%	13.8%		14.6%	17.2%		
Wednesday	16.2%	13.8%		13.7%	13.8%		
Thursday	16.3%	11.4%		15.9%	14.2%		
Friday	13.4%	12.6%		13.3%	12.5%		
Saturday	11.9%	13.8%		12.5%	14.2%		
Sunday	13.5%	17.5%		15.2%	14.1%		
Month, (%)			<0.01			<0.01	0.582
January	17.2%	30.1%		19.0%	33.6%		
February	16.4%	31.7%		16.9%	28.6%		
March	12.4%	38.2%		9.0%	37.8%		
April	0.0%	0.0%		0.0%	0.0%		
May	0.0%	0.0%		0.0%	0.0%		
June	0.0%	0.0%		0.0%	0.0%		
July	0.0%	0.0%		0.0%	0.0%		
August	0.0%	0.0%		0.0%	0.0%		
September	0.0%	0.0%		0.0%	0.0%		
October	18.1%	0.0%		18.5%	0.0%		
November	17.7%	0.0%		17.9%	0.0%		
December	18.3%	0.0%		18.8%	0.0%		

Amer.: American; DNR: do-not-resuscitate; CCM: critical care medicine; CCOT: critical care outreach team; ER: emergency room; ICU: intensive care unit; LAPS: Laboratory-based Acute Physiology Score; PA: physician assistant; SOFA: sequential organ failure assessment

* comparison between pre- and post-CCM PA on the CCOT within the same hospital

^†^ comparison between the control and intervention hospitals (data from both pre-/post-CCM PA on the CCOT combined within each hospital)

^‡^ overnight = 7pm-7am

Analysis of pre-intervention trends revealed no difference (0.2% per day change in TtT_ICU_, 95% confidence interval: -0.001%–0.3%) associated with being at the intervention versus control hospital. Similarly, there was no difference in the percent change for: (1) odds of hospital mortality (0.2% (-0.3%–0.8%)) and (2) hospital LOS (0.06% (-0.02%–0.13%)). These results support the assumption necessary for difference-in-difference analyses—that trends in outcomes for the control and intervention hospitals were similar pre-intervention.

**Fig 3:**
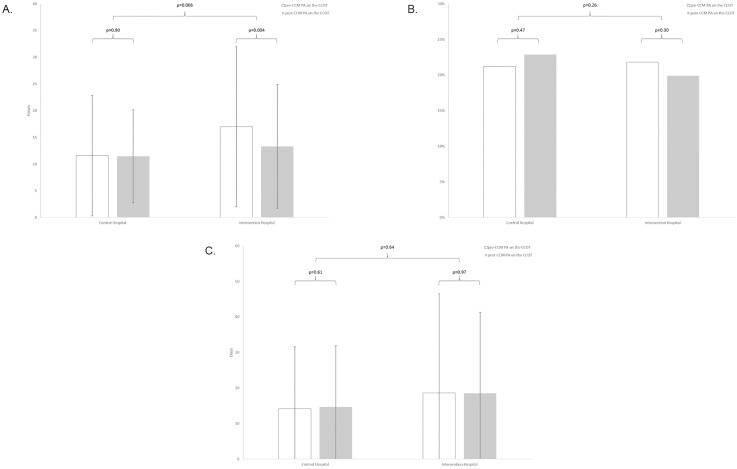
Unadjusted comparisons between intervention and control hospitals. (A) Time-to-transfer. (B) Hospital mortality. (C) Hospital length of stay.

For those patients transferred to the ICU, unadjusted TtT_ICU_ was longer in the intervention hospital (mean±sd: 17.0±15.0 hours pre- and 13.3±11.6 post-intervention vs 11.6±11.3 pre- and 11.6±8.7 post-, p = 0.006); notably, TtT_ICU_ was shorter in the post- vs pre-intervention period (p = 0.004) in the intervention but not the control hospital (p = 0.90, Fig 3). The difference-in-difference analysis demonstrated a significant 19.2% reduction in the adjusted TtT_ICU_ (95% confidence interval: 6.7%-31.6% reduction, p = 0.002) associated with adding the CCM-PA to the CCOT (Table 2).

**Table 2:** Multivariate Difference-in-Difference Analysis*.

	Time-to-Transfer^†^		Hospital Mortality		Hospital LOS	
	% change (95% CI)	p-value	OR (95% CI)	p-value	% change (95% CI)	p-value
Intervention Hospital x Post-CCM PA on CCOT	-19.2 (-31.6, -6.7)	0.002	0.76 (0.51, 1.15)	0.20	1.8 (-3.7, 7.4)	0.52
Intervention Hospital	19.0 (11.2, 26.7)	<0.001	1.15 (0.89, 1.49)	0.30	8.4 (4.8, 11.9)	<0.001
Post-CCM PA on CCOT	2.7 (-7.5, 12.9)	0.54	1.16 (0.82, 1.62)	0.41	1.1 (-3.6, 5.8)	0.63
Age^‡^	-3.1 (-4.3, 10.2)	0.008				
Ethnicity: unavailable			66.73 (4.86, 916.62)	0.002		
Insurance: private pay					-3.35 (-6.2, -0.5)	0.021
Elixhauser Comorbidity Index^§^			1.06 (1.01, 1.12)	0.024	5.44 (4.7, 6.1)	<0.001
SOFA, time of consult^§^			1.35 (1.29, 1.42)	<0.001	1.1 (0.4, 1.8)	0.002
LAPS, hospital admission^§^			1.01 (1.00, 1.01)	<0.001	-0.1 (-0.1, 0.0)	<0.001
Admitting Diagnosis: Endocrine/Metabolic/Renal					-37.5 (-74.6, -0.4)	0.047
DNR, time of consult			4.48 (1.99, 10.99)	<0.001	-14.3 (-27.8, -0.7)	0.040
Admitted from the ER			3.54 (2.46, 5.07)	<0.001		
Admitted to the ICU following consultation			0.77 (0.61, 0.99)	0.038	7.48 (4.2, 10.8)	<0.001
Consult timing: overnight					-3.30 (-6.0, -0.6)	0.016
Consult timing: month						
March	13.8 (-22.8, -4.7)	0.031			-4.32 (-8.4, -0.3)	0.036
October	-12.7 (-24.2, -1.3)	0.029				
November	-19.8 (-25.9, -4.4)	0.006				
December	-16.7 (-28.4, -4.9)	0.005				

DNR: do-not-resuscitate; CCM: critical care medicine; CCOT: critical care outreach team; ER: emergency room; ICU: intensive care unit; LAPS: Laboratory-based Acute Physiology Score; OR: odds ratio; PA: physician assistant; SOFA: sequential organ failure assessment

* all covariates listed in Table 1 were included in the multivariable models; only intervention-related variables and those with p<0.05 are provided for simplicity

^†^ Time-to-Transfer values reflect those of only patients admitted to the ICU (as others receiving CCM consultation do not have a Time-to-Transfer)

^‡^ Age <90 years compared to reference of age ≥90

^§^ Modeled as linear predictors of outcome

There was no difference in unadjusted hospital mortality between hospitals (p = 0.26) with no change pre- to post-intervention in either center (intervention hospital: 21.8% pre- vs 19.9% post-intervention, p = 0.30; control hospital: 21.2% pre- vs 22.9% post-; p = 0.47). Moreover, we found no association between CCM-PA addition to the CCOT and adjusted mortality using the difference-in-difference methodology (p = 0.20). Similarly, there was no difference in unadjusted hospital LOS between the hospitals (p = 0.64) with no change pre- to post-intervention in either center (intervention hospital, mean±sd: 18.6±28.0 days pre- vs 18.6±22.8 post-intervention, p = 0.97; control hospital: 14.2±17.4 pre- vs 14.7±17.1 post-, p = 0.61). There was also no association between CCM-PA addition to the CCOT and adjusted hospital LOS using the difference-in-difference approach (p = 0.52).

**Fig 4:**
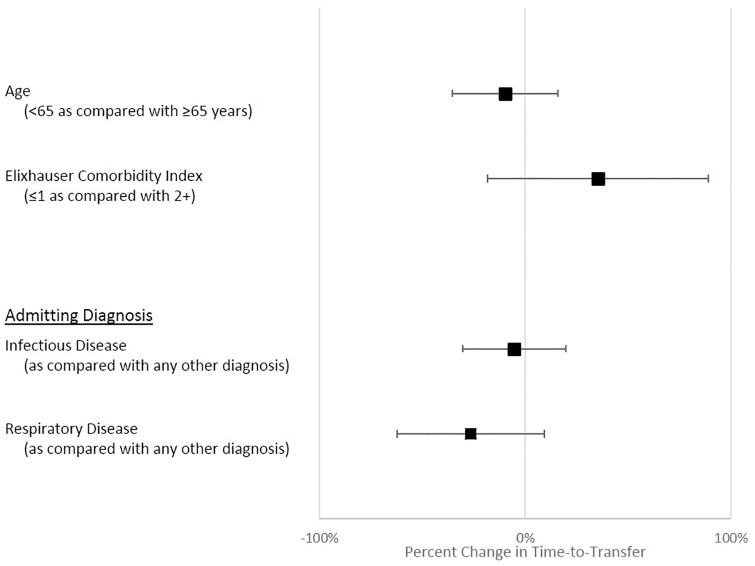
Relative impact of the introduction of the physician assistant on patient subgroups. ■ = point estimate; bars = 95% confidence interval.

Our results were consistent across subgroups. There was no association between CCM-PA addition to the CCOT and adjusted hospital mortality or LOS for patients admitted to the ICU (S1 Table). Adding the CCM-PA to the CCOT affected adjusted TtT_ICU_, hospital mortality, and hospital LOS similarly across subgroups of age, Elixhauser comorbidity burden, and infectious disease and respiratory admitting diagnoses (Fig 4).

## Discussion

Introducing a CCM-PA to the CCOT at our institution was associated with a statistically significant and clinically meaningful reduction in the TtT_ICU_ of 19.2%; or, without casemix adjustment, a 3.7 hour reduction after adding the CCM-PA (with no change at the control hospital). We were unable to identify any impact on hospital mortality or LOS, however. Interestingly, the impact on TtT_ICU_ was experienced similarly by older and younger patients, patients with high and low burden of chronic disease, and patients with different admitting diagnoses.

Our finding that adding a CCM-PA to the CCOT decreased TtT_ICU_ is novel; several factors likely drive it. First, the CCM-PA may have been the first CCOT member to evaluate patients ultimately in need of the ICU while other members were busy. The literature suggests that well-trained PAs are able to identify patients needing ICU admission; when asked to quantify patients’ likelihood of clinical deterioration, nurse practitioners and PAs anticipated patient instability as well as physicians (area under the curve for “patient acuity rating” of 0.80 for nurse practitioners/PAs vs 0.69 for medicine residents and 0.88 for attendings).[33] Thus, having these patients assessed by the CCM-PA earlier than they would be by other CCOT members could facilitate ICU transfer. Second, the CCM-PA may have been evaluating less sick patients and/or performing procedures which freed the attending or fellow to more expeditiously evaluate and activate transfers for patients in need of ICU. Finally, the CCM-PA may have identified clinical deteriorations in patients not initially thought to need the ICU on close follow-up which, had the CCM-PA not been available, would have gone unnoticed for longer. As TtT_ICU_ is defined as the time from initial request for CCOT evaluation to time of ICU arrival, this quicker identification of clinical deterioration would improve TtT_ICU_.

Our finding that CCM-PA addition to the CCOT can improve care processes without compromising outcome is consistent with published literature. While CCOTs and RR/METs are differentially staffed across institutions,[12–16] there is little data on the relative benefits of one team composition over another. The only study to directly compare staffing structures was conducted at a single academic hospital where RR/METs were led by intensivists during the daytime and medical residents overnight/on weekends.[13] Patients seen by the RR/MET during the day (versus overnight/on weekends) had similar rates of progression to cardiac arrest (1.8% vs 2.4%, p = 0.4), ICU transfer (57% vs 57%, p = 0.82), and hospital mortality (27% vs 26%, p = 0.64). Put simply, it didn’t matter who staffed the team. Furthermore, as aforementioned, nurse practitioners and PAs are as able as physicians to predict clinical deterioration.[33] These studies suggest, therefore, we can view adding a CCM-PA to our 2-person CCOT as, simply, a 50% increase in qualified personnel and capacity. Moreover, this capacity increase is achieved at approximately one-third the cost of adding an intensivist (were expansion of the team to be achieved in this way)—median CCM-PA annual salary is $90,000-$110,000 versus $275,000-$300,000 for an intensivist.[34, 35] As described by Jones *et al*., there is a dose-response curve for RR/METs activations—the more times they evaluate patients (the more patients they consult on) the better their impact on outcomes.[36] Thus, it is reasonable and consistent with published literature to observe that adding a CCM-PA to our CCOT and expanding its reach improves the CCOT’s performance. Furthermore, adding the CCM-PA may be cost effective, in terms of capacity-per-dollar.

That we did not identify an association of adding a CCM-PA to the CCOT and hospital mortality can be explained in two ways—either: (1) there is truly no impact on mortality or (2) we did not have adequate power to detect a difference which actually exists. Multiple reviews and meta-analyses have been done reaching conflicting conclusions on the impact of RR/METs on hospital mortality.[6, 8–10] It is possible, therefore, that there is truly no such impact of this intervention. Notably, however, multiple studies have reported on the negative association of delays in ICU admission (prolonged TtT_ICU_) and mortality—in the emergency department,[37] throughout the hospital,[38] post-operatively,[39] and from hospital wards [40]. In two, a delayed transfer was defined as TtT_ICU_ > 6 hours.[37, 39] We found adding the CCM-PA reduced TtT_ICU_ by an average of 3.7 hours suggesting that a subset of patients had a reduction in TtT_ICU_ of >6 hours which may also result in reducing in odds of mortality. It reasonable to suppose, therefore, that given the reduced TtT_ICU_ we saw with adding the CCM-PA to our CCOT, with a larger sample, this reduction may translate into a survival benefit. In fact, we did observe a non-significant 23.8% reduction in the odds of mortality associated with adding the CCM-PA.

Our study is the first to assess the changing composition of a CCOT and to evaluate the impact of this change on TtT_ICU_. It has limitations, however. As a single center study, more work is necessary to determine whether and how our results generalize to other institutions, especially given the variability in CCOT structure and function across hospitals as well as differences in non-CCOT resources available to identify and care for unstable patients. Given our retrospective study design, we were unable to describe the precise role the CCM-PA played for each individual patient. Their overall responsibilities were diverse; which role(s) was paramount during each encounter is not known. Moreover, we are unable to determine whether the impact of the CCM-PA was the addition of a PA in specific or, simply, the addition of a third CCOT member. Teasing out this difference will enable better determination of whether adding a CCM-PA is indeed cost-effective. If the benefits are due to the addition of a third CCOT member, cost savings related to salary differences between CCM-PAs and intensivists/other providers alone may result in cost-effectiveness; but, a full cost-effectiveness analysis is needed before this impact can be known. Moreover, if the PA brings a specific unique beneficial contribution which an intensivist would not, they may represent not only be a less costly, but also a more effective, addition. Finally, we investigated the impact of adding to the CCOT a PA with experience working in the ICU; we cannot say whether our results would be similar had PAs with different proficiencies been utilized. These limitations are balanced by our study’s strengths. First, we had data for a control hospital with similar patient population, staffing patterns, and trends in outcome pre-intervention. This allowed use of the difference-in-differences methodology to account for secular changes in practice and, thus, better evaluation of the specific impact of our intervention. This approach allows us to better control for temporal trends as well unobserved biases common across the two hospitals when compared to other single institution pre/post-implementation studies which do not have a control hospital available. Second, to address seasonal variability of critical illness both in terms of patient diagnoses/severity of illness and staffing competencies at academic centers, we compared outcomes between pre- and post-intervention periods which occurred during similar times of year.

## Conclusions

Adding a CCM-PA to our CCOT (staffed initially by an intensivist and critical care fellow) resulted in a significant improvement in patient flow without an observable impact on outcomes. Improving patient flow is, on its own, a meaningful endpoint for hospital administrators (who must report flow metrics), clinicians (who may prefer to care for critically ill patients in the ICU setting), and patients/families (who may exhibit anxiety associated with delays). Understanding how adding the CCM-PA improves flow, however, remains to be explored—does he/she simply represent an increase in capacity for the CCOT or is there something unique he/she provides to expedite throughput? Moreover, a major benefit of CCOTs would be their ability to prevent the need for ICU admission; it remains to be examined whether adding a CCM-PA to the team may assist in this goal. Critical care is a specialty in which the importance of multidisciplinary care is known. How best to utilize multidisciplinary team members in a setting like a CCOT—perhaps by targeting certain patient subgroups—is unclear. As we expand the utilization and reach of RR/METs and CCOTs, we must carefully evaluate how best to construct them to achieve their stated goals.

## Supporting Information

S1 Table Multivariate Difference-in-Difference Analysis for Patients Admitted to the Intensive Care Unit*. * all covariates listed in Table 1 were included in the multivariable models; only intervention-related variables and those with p<0.05 are provided for simplicity. † Time-to-Transfer values are identical to those in Table 2 as Time-to-Transfer includes only, by definition, patients admitted to the intensive care unit. ‡ Age <90 years compared to reference of age ≥90. § Modeled as linear predictors of outcome.
(DOCX)
