# Supplementary material for: The Impact of Adding a Physician Assistant to a Critical Care Outreach Team
Source: PLoS One. 2016 Dec 12;11(12):e0167959. doi: 10.1371/journal.pone.0167959 (PMC5152859; doi:10.1371/journal.pone.0167959)
Supplement: S1 Table — * all covariates listed in Table 1 were included in the multivariable models; only intervention-related variables and those with p<0.05 are provided for simplicity. † Time-to-Transfer values are identical to those in Table 2 as Time-to-Transfer includes only, by definition, patients admitted to the intensive care unit. ‡ Age <90 years compared to reference of age ≥90. § Modeled as linear predictors of outcome. (DOCX) [file pone.0167959.s001.docx]

S1 Table. Multivariate Difference-in-Difference Analysis for Patients Admitted to the Intensive Care Unit.*

|  | Time-to-Transfer^†^ | | Hospital Mortality | | Hospital LOS | |
| --- | --- | --- | --- | --- | --- | --- |
|  | % change  (95% CI) | p-value | OR  (95% CI) | p-value | % change  (95% CI) | p-value |
| Intervention Hospital x Post-CCM PA on CCOT | -19.2  (-31.6,-6.7) | 0.002 | 1.16  (0.47, 2.86) | 0.75 | 5.1  (-6.8,16.9) | 0.40 |
| Intervention Hospital | 19.0  (11.2, 26.7) | <0.001 | 0.78  (0.44, 1.36) | 0.36 | 3.9  (-3.5, 11.3) | 0.30 |
| Post-CCM PA on CCOT | 2.7  (-7.5, 12.9) | 0.54 | 0.95  (0.45, 2.01) | 0.90 | -7.4  (-17.1, 2.4) | 0.14 |
| Age^‡^ | -3.1  (-4.3, 10.2) | 0.008 |  |  |  |  |
| Race: Black/African American |  |  | 0.10  (0.00, 0.86) | 0.036 |  |  |
| Race: Asian |  |  | 0.06  (0.00,0.88) | 0.041 |  |  |
| Elixhauser Comorbidity Index^§^ |  |  | 1.20  (1.08, 1.34) | 0.001 | 5.6  (4.1, 7.0) | <0.001 |
| SOFA, time of consult^§^ |  |  | 1.41  (1.27, 1.57) | <0.001 |  |  |
| LAPS, hospital admission^§^ |  |  |  |  | -0.2  (-0.3, -0.1) | 0.001 |
| Admitting Diagnosis |  |  |  |  |  |  |
| Cardiovascular |  |  | 0.06  (0.01, 0.28) | <0.001 |  |  |
| Endocrine/Metabolic/Renal |  |  | 0.09  (0.02, 0.36) |  |  |  |
| Gastrointestinal |  |  | 0.11  (0.03, 0.43) |  |  |  |
| Infectious Disease |  |  | 0.21  (0.07, 0.64) |  |  |  |
| Neurologic |  |  | 0.15  (0.04, 0.57) |  |  |  |
| Respiratory |  |  | 0.06  (0.02, 0.24) |  |  |  |
| Consult timing: Thursday |  |  | 0.25  (0.10,0.63) | 0.003 |  |  |
| Consult timing: month |  |  |  |  |  |  |
| March | 13.8  (-22.8, -4.7) | 0.031 |  |  |  |  |
| October | -12.7  (-24.2, -1.3) | 0.029 |  |  |  |  |
| November | -19.8  (-25.9, -4.4) | 0.006 |  |  |  |  |
| December | -16.7  (-28.4, -4.9) | 0.005 |  |  |  |  |

CCM: critical care medicine; CCOT: critical care outreach team; LAPS: Laboratory-based Acute Physiology Score; OR: odds ratio; PA: physician assistant; SOFA: sequential organ failure assessment

* all covariates listed in Table 1 were included in the multivariable models; only intervention-related variables and those with p<0.05 are provided for simplicity

† Time-to-Transfer values are identical to those in Table 2 as Time-to-Transfer includes only, by definition, patients admitted to the intensive care unit

‡ Age <90 years compared to reference of age ≥90

§ Modeled as linear predictors of outcome
